# Tricuspid Valve Replacement in a Patient with a Leadless Cardiac Pacemaker: Current Guidelines and Recommendations for Perioperative Management

**DOI:** 10.1155/2021/5559830

**Published:** 2021-07-01

**Authors:** River Hames, J. W. Awori Hayanga, Diane Schmidt-Krings, Timothy Goldhardt, John Bozek, Donald Siddoway, Stanley Schmidt, John Lobban, Heather K. Hayanga

**Affiliations:** ^1^West Virginia University School of Medicine, 1 Medical Center Drive, Morgantown, WV 26506, USA; ^2^Department of Cardiovascular and Thoracic Surgery, West Virginia University, 1 Medical Center Drive, Morgantown, WV 26506, USA; ^3^Department of Anesthesiology, West Virginia University, 1 Medical Center Drive, Morgantown, WV 26506, USA; ^4^Division of Cardiovascular and Thoracic Anesthesiology, West Virginia University, 1 Medical Center Drive, Morgantown, WV 26506, USA; ^5^Division of Cardiology, West Virginia University, 1 Medical Center Drive, Morgantown, WV 26506, USA

## Abstract

Leadless cardiac pacemakers were developed to reduce complications associated with conventional transvenous pacemakers. While this technology is still relatively new, devices are increasingly being implanted. The perioperative management of patients with these devices has been underreported; we thus seek to add to the limited body of knowledge of perioperative management of patients with leadless cardiac pacemakers. An elderly female patient with a Micra VR transcatheter pacing system leadless cardiac pacemaker placed for tachycardia-bradycardia syndrome with intermittent complete heart block was scheduled for elective tricuspid valve replacement for severe tricuspid regurgitation. Pacemaker interrogation was performed several hours prior to the scheduled surgery based on the electrophysiologist's availability; the device was kept in its programmed VVIR mode, and the base rate was increased from 60 to 80 beats per minute in anticipation of the upcoming surgery. Upon preoperative evaluation, the anesthesiologist asked that the electrophysiology team be placed on standby intraoperatively due to the concern that either oversensing in the setting of pacemaker dependence and/or undesirable tachycardia from rate-responsive pacing could occur. The surgeon used monopolar electrocautery for the duration of the cardiac surgery. Despite the patient having evidence of pacemaker dependence in the intensive care unit preoperatively, no electromagnetic interference leading to oversensing nor rate modulation was detected during intraoperative electrocardiographic and intraarterial invasive monitoring. Evidence-based guidelines regarding perioperative management specifically of leadless cardiac pacemakers do not exist. As these devices become more prevalent, further evaluation will be paramount to determine whether existing guidelines for perioperative management of conventional transvenous pacemakers apply.

## 1. Background

Since the first successful implant in 2012, increasing numbers of leadless cardiac pacemakers (LP) have been placed for indications such as symptomatic bradycardia [1]. The Micra VR transcatheter pacing system (TPS) from Medtronic was approved by the US Food and Drug Administration (FDA) in April 2016 [2]. LP technology reduces complications associated with conventional transvenous pacemakers such as lead dislodgement, lead fracture, and pocket infections [3]. This case report will focus on the anesthetic management of a patient with the Micra VR undergoing cardiac surgery. The Micra VR is a single-chamber leadless cardiac pacemaker. Conventional transvenous pacemakers consist of a subcutaneous electrical generator and insulated leads that implant into the endocardium via the subclavian vein and superior vena cava. The Micra VR is placed transvenously through the femoral vein and is directly implanted into the right ventricle using 4 electrically inactive nitinol tines near the apex of the heart, ideally on the septum [2, 4].

Reynolds et al. published in 2016 that the safety and efficacy of the Micra VR exceeded prespecified goals with low and stable pacing thresholds [2]. The purpose of this device is to eliminate the need for a pacemaker pocket and transvenous leads that are common sources of complications in conventional transvenous pacemakers. The Micra VR leadless pacemaker is a viable alternative to transvenous pacemakers in patients indicated for single-chamber ventricular demand (VVI) pacing leading to the devices becoming commonplace in anesthesia practice [1]. Thus far, only other case reports have been the source of guidance for intraoperative management of these devices. The American Society of Anesthesiologists “Practice Advisory for the Perioperative Management of Patients with Cardiac Implantable Electronic Devices: Pacemakers and Implantable Cardioverter–Defibrillators 2020” (ASA CIED) lacks specific recommendations regarding LPs [5]. Therefore, we seek to present a patient with a Micra VR who required cardiac surgery to describe our intraoperative management as well as review the current literature to summarize appropriate perioperative management of leadless cardiac pacemakers.

## 2. Case Report

An 82-year-old female with a history of coronary artery disease status postcoronary artery bypass grafting in 1997 and coronary stents in 2008, congestive heart failure, cerebrovascular accident, prior episodes of gastrointestinal bleeding thought to be secondary to chronic anticoagulation for atrial fibrillation, and tachycardia-bradycardia syndrome with intermittent complete heart block requiring Micra VR placement in September 2017 was found to have symptomatic severe tricuspid regurgitation in June 2018. She was scheduled to undergo an elective tricuspid valve replacement and closure of an incidentally discovered patent foramen ovale.

The Micra VR had been interrogated 3 months prior to cardiac surgery; however, pacemaker interrogation was performed several hours prior to the scheduled surgery based on the electrophysiologist's availability. The device was kept in its programmed VVIR mode, and the rate was increased from 60 to 80 beats per minute in anticipation of the upcoming surgery. Upon subsequent preoperative evaluation by the anesthesiologist, electrocardiography showed evidence of pacemaker dependence. The anesthesiologist asked that the electrophysiologist be placed on standby intraoperatively due to the concern that either oversensing in the setting of pacemaker dependence and electromagnetic interference (EMI) and/or undesirable tachycardia from rate-responsive pacing could occur.

**Figure 1:**
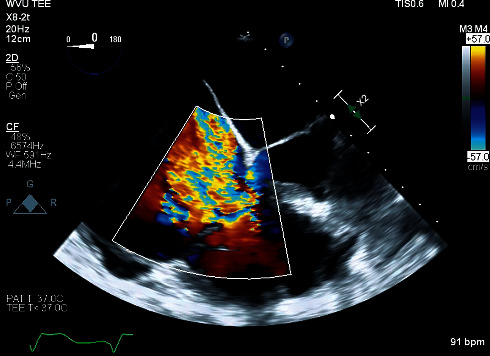
Transesophageal echocardiography, midesophageal four-chamber view demonstrating severe tricuspid regurgitation in patient with a leadless pacemaker.

**Figure 2:**
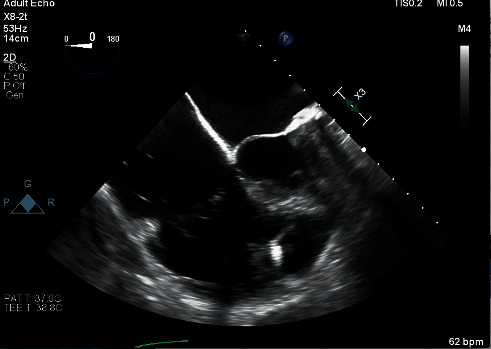
Transesophageal echocardiography, modified midesophageal four-chamber view demonstrating a leadless pacemaker in the right ventricle.

After standard induction of general anesthesia, severe tricuspid regurgitation was observed once again using transesophageal echocardiography (TEE) (Figure 1), and the Micra VR was evident during the TEE evaluation of the right ventricle (Figure 2). The patient underwent a right-sided thoracotomy with tricuspid valve replacement using a bioprosthetic valve. The surgeon used monopolar electrocautery for the duration of the cardiac surgery. No EMI leading to oversensing nor rate modulation was detected during intraoperative electrocardiography and interarterial invasive monitoring despite the operative proximity to the Micra VR and use of monopolar electrocautery. The operation was uneventful, and the patient was transported to the intensive care unit intubated, on epinephrine, norepinephrine, and milrinone postoperatively. Later that day, she was noted to not be moving her left side; emergent head computed tomography (CT) was significant for right-sided edema. Repeated head CT after continued weakness showed severe diffuse edema. Given these findings, the family decided to proceed with comfort care measures; she died on postoperative day eleven.

## 3. Discussion

Each year, over 250,000 pacemakers are implanted in the United States, primarily to treat bradycardic rhythms [6]. Conventional transvenous pacemakers were introduced in the 1960s, and over the years, the technology has improved, but complications tied to the transvenous leads and pacemaker pocket for the subcutaneous electrical generator remain [7]. The leads increase the risk of infection or myocardial or lung injury [7]. Other potential complications include infection of the subcutaneous pocket, hematoma, extrusion, and scarring. One-tenth of patients receiving a conventional transvenous pacemaker will have acute and chronic lead-associated complications [8]. This may necessitate lead replacement, subjecting the patient to more surgeries. Conventional pacemakers have been shown to have 20% (11% lead, 8% pocket) complication rate at 5 years [2]. Even for complication-free transvenous pacemaker implants, it can be distressing for the patient to have a subcutaneous bulge, reminding them of their dependence on a pacemaker. Contrastingly, patients with LPs have been shown to have superior physical function, mental health, and significantly lower pacemaker-related discomfort [9]. Indications for leadless pacing include atrioventricular (AV) block with atrial fibrillation, bradycardia, and AV block or sinus node disease that does not require frequent ventricular pacing [1, 10].

## 4. Micra VR

The Micra VR is limited in function by only being capable of single-chamber pacing. Single-chamber pacing devices make up less than 10% of implants [7]. In addition to being limited by narrow indications for a pacemaker, the Micra VR cannot produce atrioventricular synchrony to reduce the risk of pacemaker syndrome compared to conventional pacemakers [7]. The device has not been studied in patients with a mechanical tricuspid valve, an implanted vena cava filter, or left ventricular assist device [2]. The Micra VR can be remotely interrogated via the CareLink system [11]. It is 1.5 T and 3 T MRI conditionally compatible and allows for rate-responsive pacing and automated pacing capture threshold management [1]. Furthermore, Reynolds et al. demonstrated the Micra VR had significantly fewer major complications compared to a historical cohort of patients that received transvenous pacers [2]. In a prospective survey on choosing an LP or conventional transvenous pacemaker, LPs were favored in patients with high risk of infection, low rate of ventricular pacing, and in those more likely to have atrial fibrillation [12]. Mitigation of infection risk was exemplified in a study that removed a conventional transvenous pacemaker due to active infection and concurrently implanted an LP [13].

The Micra VR device is introduced via the femoral vein. The device requires a 23 French introducer resulting in a 0.7–0.75% rate of access-related complications at implantation [14, 15]. Recently, it was shown that a single tapered Coons dilator could be substituted, reducing surgical time [14]. Others have shown that an open approach is possible for device implantation [16]. Potential complications may arise with multiple implantation attempts by damaging vessels and myocardium potentially leading to pericardial effusion [2, 4, 17]. Thus, inadequate femoral venous anatomy limits the feasibility of implantation [7, 18]. A recent study demonstrated 43% of patients implanted with a LP at one year of follow-up were graded with more severe tricuspid valve regurgitation compared to baseline. Of note, this increase of tricuspid regurgitation was not statistically significantly different from the transvenous pacemaker control group. Additionally, septal positioning of the LP increased tricuspid regurgitation compared to apical positioning [19]. These results potentially explain the degree of tricuspid regurgitation observed in this patient. Patients with small body habitus and decreased right ventricular size may increase difficulty in retrieving these devices if removal is deemed necessary [20].

The Micra VR attaches to the right ventricular septal wall with fixed nitinol tines. This design provides the device with the advantage of no device pocket and no leads [2, 4]. Only two nitinol tines are needed for implantation, yet the device is equipped with four to reduce the risk of dislodgement. Encapsulation of the device over time should further reduce the risk of dislodgement, but the degree of encapsulation around the device is unpredictable [21]. Additionally, device attachment to the myocardium may cause inflammation which has been proposed to increase the risk of ventricular arrhythmias [22]. Aparisi et al. reported on three cases of a temporal relationship of Micra VR implantation and the subsequent development of malignant ventricular arrhythmias. Aparisi et al. concluded further study into ventricular arrhythmias after LP implantation is warranted [22].

The Medtronic manual for Micra VR recommends avoiding electrosurgery in patients with an LP when feasible due to possible oversensing, unintended tissue damage, tachyarrhythmias, device damage, or device malfunction. When electrosurgery cannot be avoided, the manual recommends bipolar electrocautery, Medtronic Advanced Energy surgical incision technology, or Hyfrecator use over monopolar electrocautery [23]. Medtronic Advanced Energy surgical incision technology utilizes radiofrequency to generate electrical plasma at lower temperatures compared to traditional electrocautery [24]. A Hyfrecator is a low-powered electrocautery device that works by emitting low-power high-frequency high-voltage AC electrical pulses [25]. If monopolar electrocautery is used, the manual suggests that the electrical current pathway should not pass through or within 15 cm of the LP and recommends avoiding using monopolar electrocautery within 15 cm of the device. The manual further suggests to mitigate the effects of oversensing, if patient appropriate, by programming the LP to the asynchronous (VOO) mode [23].

Although this case predated the ASA CIED, this practice advisory is congruent with the preexisting Heart Rhythm Society/American Society of Anesthesiologists expert consensus statement from 2011 for perioperative management of implantable defibrillators and pacemakers; both recommend to alter the pacing mode in pacemaker-dependent patients and to suspend the active sensor for rate-responsive pacing with any type of CIED when monopolar electrocautery is used superior to the umbilicus [5, 26]. However, neither practice advisory nor consensus statement specifically discusses LP management; the latter predated the first successful LP implantation. In this case, given that the electrophysiologist had already seen the patient several hours prior to surgery, was not readily available, and guidelines did not exist specifically for perioperative LP management, the plan was for the electrophysiologist to come to the operating room to reprogram the LP to VOO asynchronous pacing at 80 beats per minute if significant EMI with evidence of insufficient perfusion using arterial line monitoring or undesirable tachycardia from rate-responsive pacing was evident. However, this plan was not implemented, as there was no evidence of EMI causing oversensing despite pacemaker dependence nor unwarranted tachycardia. Therefore, the LP remained in the VVIR mode, while monopolar electrocautery was used superior to the umbilicus and within 15 cm of the device.

Evidence-based guidelines specific to perioperative management of LPs are lacking. Other case reports have also identified the lack of clinical data for perioperative management of LPs and call for further study [27]. This highlights the need for more studies investigating outcomes under various surgical and anesthetic management conditions of patients with LPs. ASA CIED only mentions LPs in the footnotes when generalized guidelines for all CIEDs are provided, stating that a magnet may not elicit a response with some LPs. Thus, the only recommendations provided are for CIEDs in general and include a generalized preoperative evaluation, placing the CIED into asynchronous mode if EMI is likely in the pacemaker-dependent patient undergoing surgery above the umbilicus, using bipolar electrocautery or ultrasound scalpel when feasible, and continuously monitoring the patient [5].

## 5. Next Steps

As of now, LP implantation with Micra VR is limited only to patients who are candidates for single-chamber ventricular pacing. In addition to other manufacturers developing similar technologies, we expect the capability of this technology to expand and to have the functionality of conventional pacemakers, making their prevalence more robust. For example, in 2020, the FDA approved Medtronic's expanded indications for LPs that involves an algorithm for redesigned Micra device software called Micra AV to treat atrioventricular heart block [28]. For this reason, we also expect increasing numbers of patients with these devices implanted requiring surgery. Until evidence-based criteria are created, we recommend following the current ASA CIED where feasible but also taking into consideration the Medtronic clinician manual as well. This clinician manual provides additional warnings and guidelines (Table 1). One case report suggests utilizing TEE before and after surgery to verify device position and to minimize right ventricle manipulation to decrease risk of dislodgement [29]. Another highlights the importance of preoperative assessment of the LP device and having the electrophysiology service on standby with alternative pacing options readily available [30]. For patients undergoing retrieval of an LP, use of heparin is advised, and the difficulty of retrieval largely depends on the device position compared to the varying degrees of fibrosis around the device [31].

Further discussion on EMI during surgery in patients with LPs is warranted. EMI is very common in the operating room, and options other than monopolar cautery may not be a surgical option. While appropriate precautions should be undertaken, further evaluation needs to occur to determine whether LPs are perhaps potentially more resistant to EMI or may require separate practice advisory recommendations. In this case report, no EMI causing oversensing from monopolar electrocautery used within 15 cm of the device was observed in a patient with pacemaker dependence undergoing cardiac surgery. In conclusion, leadless cardiac pacemakers may have advantages over transvenous pacemakers, although further investigation and long-term studies are needed. Regardless, these devices will likely become more common in surgical patients.

## Figures and Tables

**Table 1:** Highlights of warnings, precautions, and guidance for clinicians performing medical procedures on cardiac device patients, modified from Medtronic manual for Micra VR.

Ablation	Ablation is a surgical technique in which radio frequency or microwave energy produces thermal energy to destroy tissues. Ablation used in cardiac device patients increases the risk of induced ventricular tachyarrhythmia, device over sensing, unintended tissue damage, device damage, or device malfunction. The Micra VR is designed to withstand exposure to ablation energy. Medtronic recommends the following to mitigate these risks: (i) Ensure that temporary pacing and defibrillation equipment is available. (ii) Avoid the direct contact between the ablation catheter and the Micra VR (iii) Position the return electrode patch, so that the electrical energy path does not pass through or near device. (iv) Monitor the patient during ablation with at least 2 separate methods, such as arterial pressure, ECG, taking patient's pulse, pulse oximetry, or Doppler pulse detection. To prevent the effects of over sensing, program the Micra VR to the asynchronous pacing mode (e.g., VOO) if patient appropriate. After ablation, restore device parameters.

Diagnostic radiology	Diagnostic radiology includes the following: (i) Computerized axial tomography (CT or CAT scan) (ii) Fluoroscopy (iii) Mammograms (iv) X-rays Diagnostic radiology even at accumulated doses is not enough to damage the Micra VR. Precautions should be taken if the device is directly in the beam of radiation for CT scans. Similar interference may be observed in high-intensity fluoroscopy. Oversensing may occur for the duration of time the device is in the beam. Medtronic recommends placing the Micra VR into the asynchronous pacing mode (e.g., VOO), if patient appropriate, when the device will be directly in the CT scan beam for longer than 4 seconds. This is to avoid or mitigate the effects of oversensing. Restore device parameters upon completion of scan.

Diagnostic ultrasound	Diagnostic ultrasound is a noninvasive imaging method to visualize internal anatomy and measure heart rates or blood flow. Echocardiogram, a form of diagnostic ultrasound, which is directed at cardiac tissue, poses no risk of electromagnetic interference.

Electrosurgery	Electrosurgery (including electrocautery, electrosurgical cautery, Medtronic Advanced Energy surgical incision technology, and Hyfrecator) is a process in which electric energy generated by a probe is used to control bleeding and cut tissue. Electrosurgery used on cardiac device patients increases the risk of device oversensing, unintended tissue damage, tachyarrhythmias, device damage, or device malfunction. Medtronic provides the following recommendations when electrosurgery cannot be avoided: (i) Have temporary pacing and defibrillation equipment available on standby. (ii) Use a bipolar electrosurgery system or Medtronic Advanced Energy surgical incision technology or Hyfrecator, if possible before considering the use of monopolar electrosurgery. If a monopolar electrosurgery is used, position the return electrode patch, so that the electrical current pathway does not pass through or within 15 cm of the Micra VR. (i) Do not apply monopolar electrosurgery on tissues within 15 cm of the device. (ii) Use short, intermittent, and irregular bursts at the lowest clinically appropriate energy levels possible (iii) Monitor the patient during ablation with at least 2 separate methods, such as arterial pressure, ECG, taking patient's pulse, pulse oximetry, or Doppler pulse detection. To prevent the effects of over sensing, program the Micra VR to the asynchronous pacing mode (e.g., VOO) if patient appropriate. After surgery, restore device parameters and interrogate device function.

External defibrillation and cardioversion	External defibrillation and cardioversion deliver an electrical shock to the heart to convert abnormal heart rhythms to a sinus rhythm. The Micra VR is designed to withstand exposure to external defibrillation and cardioversion. Damage to Micra VR by external shock is still possible, especially with increasing energy levels. Device interrogation is recommended following external defibrillation or cardioversion.

Magnetic resonance imaging (MRI)	The Micra VR is 1.5 T and 3 T MR conditional when specific criteria are met. The following criteria are from the Micra MRI Technician Manual: Cardiology requirements (i) No abandoned leads can be present (ii) Pacing amplitude is ≤ 4.5 V at the programmed pulse width. (iii) No diaphragmatic stimulation is observed when MRI SureScan is programmed to on. (iv) The SureScan device that is beyond its projected service life is programmed to device off. Radiology requirements (i) The MRI has a maximum spatial gradient of ≤25 T/m (2500 gauss/cm) (ii) Gradient systems with maximum gradient slew rate performance per axis of ≤ 200 Tesla per meter per second (T/m/s) (iii) The whole body averaged specific absorption rate (SAR) must be ≤ 4.0 W per kilogram (W/kg). The head SAR must be ≤ 3.2 W/kg. Continuously monitor the patient's hemodynamic status and have an external defibrillator available. Potential adverse outcomes in the MR environment: (i) Potential for VT/VF induction when the patient is programmed to an asynchronous pacing mode during MRI SureScan (ii) Damage to the device causing the device to fail to detect or treat irregular heartbeats or causing the device to treat the patient's condition incorrectly

## Data Availability

No data were used to support this study.
